# Genetic Diversity, Virulence Factors and Antibiotic Resistance of *Listeria monocytogenes* from Food and Clinical Samples in Southern Poland

**DOI:** 10.3390/pathogens13090725

**Published:** 2024-08-27

**Authors:** Anna Żurawik, Tomasz Kasperski, Aldona Olechowska-Jarząb, Paulina Szczesiul-Paszkiewicz, Iwona Żak, Michał Wójcicki, Elżbieta Maćkiw, Agnieszka Chmielarczyk

**Affiliations:** 1 Faculty of Medicine, Jagiellonian University Medical College, Czysta 18 Str., 31-121 Cracow, Poland; anna.zurawik@student.uj.edu.pl (A.Ż.); paulina.szczesiul-paszkiewicz@student.uj.edu.pl (P.S.-P.); 2 Department of Microbiology, Faculty of Medicine, Jagiellonian University Medical College, Czysta 18 Str., 31-121 Cracow, Poland; tomasz.kasperski@doctoral.uj.edu.pl; 3 Department of Pharmaceutical Microbiology, Faculty of Pharmacy, Jagiellonian University Medical College, Medyczna 9 Str., 30-688 Cracow, Poland; aldona.olechowska-jarzab@uj.edu.pl; 4 Department of Microbiology, University Hospital, Jakubowskiego 2 Str., 30-688 Cracow, Poland; 5 Department of Clinical Microbiology, University Children’s Hospital of Krakow, Wielicka 256 Str., 30-663 Cracow, Poland; izak@usdk.pl; 6 Department of Microbiology, Prof. Waclaw Dabrowski Institute of Agricultural and Food Biotechnology—State Research Institute, Rakowiecka 36 Str., 02-532 Warsaw, Poland; michal.wojcicki@ibprs.pl; 7 Department of Food Safety, National Institute of Public Health NIH—National Research Institute, Chocimska 24 Str., 00-791 Warsaw, Poland; emackiw@pzh.gov.pl

**Keywords:** *Listeria monocytogenes*, clinical samples, food samples, virulence, resistance, pulsed-field gel electrophoresis

## Abstract

Listeriosis is one of the most serious foodborne diseases under surveillance, with an overall mortality rate in the EU currently being high at 18.1%. Therefore, this study aims to investigate *Listeria monocytogenes* strains isolated from clinical and food samples for susceptibility to antimicrobials, presence of virulence factors, and genetic diversity. Species were identified using the MALDI-TOF, resistance to 11 antibiotics was determined according to EUCAST guidelines, and multiplex PCR was used for serotyping and detecting virulence genes. Strains were genotyped using the PFGE method. Clinical strains showed full sensitivity to all tested antibiotics. In total, 33.3% of strains from food products were found to be resistant to ciprofloxacin and 4.2% to tetracycline. Most of the tested isolates (79.2%) belonged to serotype 1/2a-3a, and the rest (20.8%) belonged to serotype 4ab-4b,4d-4e. Five virulence genes (*prfA*, *hlyA*, *plcB*, *inlA*, and *lmo2672*) were detected in all strains studied. The *llsX* gene was the least common, in 37.5% of clinical strains and 18.75% of strains isolated from food products. Among the analyzed strains, 13 strains displayed unique PFGE profiles. The other 11 strains belong to 3 clusters of pulsotypes: cluster 1 (2 strains), cluster 2 (6 strains), and cluster 3 (2 strains). The percentage of hospitalizations and deaths of Polish patients with listeriosis indicates the seriousness of this disease, especially in an aging society, while the molecular testing of clinical strains has been rarely performed, which makes it difficult to determine the source of infection.

## 1. Introduction

*Listeria monocytogenes* is a Gram-positive rod-shaped bacteria occurring in the environment, primarily in water, soil, and plants. It is a facultatively anaerobic, psychrotolerant pathogen, able to grow at various temperatures (1–45 °C), with the optimal temperature being in the range of 30–37 °C [1]. The main source of *L. monocytogenes* infection for humans is food, including unpasteurized milk and dairy products, soft cheese, raw meat, sausages, smoked fish, vegetables, frozen foods, and ready-to-eat (RTE) dishes. *L. monocytogenes* is a significant foodborne pathogen responsible for listeriosis, which can manifest as sporadic infections or outbreaks and has a substantial global mortality rate of 20–30% [2,3].

Symptoms of listeriosis may be mild such as diarrhea and low-grade fever, but the disease can also lead to invasive infection including meningitis and encephalitis, bacteremia, and its complications such as endocarditis [4,5]. Those most vulnerable to infection include the elderly, pregnant women, newborns, and immunocompromised individuals, particularly those with cancer or chronic diseases [6].

Listeriosis ranks as the fifth most commonly reported zoonosis in humans within the European Union and is among the most serious foodborne diseases monitored by EU authorities. It has the highest hospitalization rate among all zoonotic diseases [7]. In 2022, the notification rate in the EU reached 0.62 cases per 100,000 population, marking a 15.9% increase compared to 2021, and representing the highest rate and number of cases since 2007 [7]. The highest incidence was observed in individuals over 64 years old, with 2.1 cases per 100,000 population. Denmark, Finland, and Sweden reported the highest incidence rates in Europe [8]. In Poland, listeriosis has been a mandatory notifiable disease since 1963. In 2023, 242 cases of listeriosis were recorded, and this is a noticeable increasing trend; in 2022, there were 142 cases, and in 2015—70, cases [9].

In 2022, the overall European Union case fatality rate was high (18.1%), higher than in 2021 and 2020 (13.7% and 13.0%, respectively) [7]. In addition to the high mortality rate, it is also worrying that *L. monocytogenes* exposed to sublethal concentrations of antimicrobials may develop resistance and pose a therapeutic problem [10].

Although *Listeria* species are susceptible to a wide range of antimicrobial agents, they have intrinsic or natural resistance to a select number of antimicrobial compounds. Natural resistance varies among the *Listeria* genus, but all *Listeria* species tested, including *L. monocytogenes*, were sensitive or indirectly resistant aminoglycosides, tetracyclines carbapenems, chloramphenicol, dalfopristin/quinupristin, glycopeptides, lincosamides, macrolides, cefotiam, cefoperazone, first- and second-generation cephalosporins (cefaclor, cefazolin, loracarbef), and penicillins (except for oxacillin). However, multidrug resistance in *L. monocytogenes* was first described as early as 1988 in France [11]. While *L. monocytogenes* is generally considered to retain a high susceptibility to most clinically relevant antimicrobials (such as β-lactams, either alone or combined with aminoglycosides), the widespread use of antibiotics and the potential for cross-resistance to drugs or chemotherapeutics used as growth promoters in animal production could lead to the spread of antibiotic resistance among these strains [12].

The highest proportions of *L. monocytogenes* in food products and food industry were observed for fish (2.6%), fishery products (2.5%), and products of meat origin other than fermented sausages (2.5%) [7]. Research conducted in Poland showed contamination of meat products at the level of 2.1% [13], fish at 5.8% [14], vegetables at 0.56% [15], and RTE products at 0.3% [16]. *L. monocytogenes* infections cannot always be linked to a food source, but foodborne outbreaks are a problem and cause a large number of hospitalizations and deaths. There are also epidemics with a large geographical range. In 2022, 35 foodborne outbreaks with 296 cases of illness were identified. Among them, 17 were designated as strong-evidence outbreaks with implicated food vehicle pig meat and products (5 outbreaks), fish and fish products (4 outbreaks), mixed food (3 outbreaks), vegetables and juices and other products (2 outbreaks), and dairy products other than cheeses (2 outbreaks) [7]. Additionally, four multi-country *L. monocytogenes* outbreaks were identified. For two events, the probable sources were processed meat products, while salmon and almond milk cheese were probable sources for one event each [7]. The whole-genome sequencing (WGS) method plays the most important role in epidemiological investigations, as it allows for the rapid detection of *L. monocytogenes* clusters in patients who are suffering from an infection with the same strain. In many countries, WGS is used routinely, e.g., in Switzerland [17], but the pulsed-field gel electrophoresis (PFGE) method is still used during epidemic investigations in many places [18,19]. In addition to determining clones, the serological identification of *L. monocytogenes* strains isolated from food is also important because specific serotypes show different virulent properties and the ability to survive in food products. Approximately 90–95% of strains isolated from contaminated food, animal, and human samples fall into serotypes 1/2a, 1/2b, 1/2c, and 4b. Serotypes 1/2a and 1/2b are most frequently found in food samples, whereas serotype 4b is predominantly associated with clinical cases and is commonly linked to outbreaks [13,20,21,22,23,24].

Many virulence factors produced by *L. monocytogenes* play an important role in establishing infections. *prfA* and *lmo2672* genes encode important transcription-regulating elements, while *inlA*, *inlC*, and *inlJ* genes encode internal adhesion and invasion of *L. monocytogenes* into the human intestinal epithelial cells. Internalin A is a species-specific surface protein that plays an essential role in pathogen entry into host cells, internalin C contributes to the post-intestinal stages of *L. monocytogenes* infection, and internalin J is a leucine-rich repeat (LRR) protein that is structurally related to the pathogen invasion factor. Phospholipases PlcA and PlcB promote the rupture of the phagocytic vacuole. Furthermore, listeriolysin S encoded by a group of *lls* genes shows bactericidal activity and plays a crucial role in the survival of *L. monocytogenes* [25,26,27,28,29].

The objective of the present study was to investigate *Listeria monocytogenes* isolated from food matrices and clinical samples (blood, cerebrospinal fluid) for susceptibility to various antimicrobial agents, the presence of virulence factors, and genetic diversity.

## 2. Materials and Methods

### 2.1. Clinical Strains

Clinical strains of *L. monocytogenes* (*n* = 8) were collected in cooperation with the microbiological laboratory of the University Hospital in Krakow and the laboratory of the University Children’s Hospital of Krakow. Seven strains came from adults’ bloodstream infections (BSI), and one strain came from cerebrospinal fluid from a child.

### 2.2. Food-Based Strains

In 2023, 124 food products purchased in retail outlets were tested. These were 56 samples taken from dumplings with meat, 9 from pielmieni, 4 from small Polish ravioli with meat, 4 from ravioli with cheese, 26 samples of smoked salmon, and 25 samples of camembert cheese. Food products were purchased within their best-before date (a day or two before the end of the best-before date). They were transported for a maximum of 2 h from shops to the microbiological laboratory and immediately subjected to analysis.

Moreover, in cooperation with the Department of Food Safety at the National Institute of Public Health—National Institute of Hygiene—National Research Institute (NIPH-NIH; Warsaw, Poland), 10 strains of *L. monocytogenes* were collected from food products tested in 2022. Furthermore, in cooperation with the Department of Microbiology at the Prof. Wacław Dąbrowski Institute of Agricultural and Food Biotechnology—State Research Institute (IAFB; Warsaw, Poland), 2 strains of the *L. monocytogenes* were collected from food products tested in 2020.

In this study, *L. monocytogenes* ATCC 35152 and *L. monocytogenes* ATCC 19115 were used as reference strains.

All isolated and collected strains were stored in the Department of Microbiology, Jagiellonian University Medical College, Krakow, at −70 °C using Microbank^®^ (Biomaxima, Lublin, Poland).

### 2.3. Microbiological Culture

The food products were tested for the presence of *L. monocytogenes* according to PN-EN ISO 11290-1:2017-07 [30]. Briefly, food samples weighing 25 g were placed in a stomacher bag and homogenized with 225 mL of Half Fraser Broth medium (BTL, Łódź, Poland) for 2 min. Then, the samples were incubated at 30 °C for 24 h. Next, 1 mL of the culture was transferred to 10 mL of the Fraser Broth with the addition of Fraser Selective Supplement (BTL, Łódź, Poland) and incubated at 37 °C for 48 h. The culture was then streaked onto the Palcam and ALOA agar plates (BTL, Łódź, Poland). Typical colonies growing on these media were selected and frozen for further identification.

### 2.4. Species Identification

All strains, both from food sample cultures and collected from collaborating laboratories, were identified using MALDI-TOF (Matrix-Assisted Laser Desorption Ionization Time-of-Flight Mass Spectrometry) VITEK MS (bioMérieux, Lyon, France).

### 2.5. Antimicrobial Susceptibility Testing

*L. monocytogenes* isolates were tested in vitro for their susceptibility to 11 antimicrobial agents (Oxoid, Hampshire, UK). Antimicrobial susceptibility tests were performed using the Kirby–Bauer disk diffusion method according to the European Committee on Antimicrobial Susceptibility Testing (EUCAST) standards on Mueller–Hinton agar with an addition of 5% defibrinated horse blood (Oxoid, Basingstoke, UK). The inoculum of each *L. monocytogenes* strain was 0.5 McFarland. The plates were incubated at 37 °C for 18 ± 2 h. The following antimicrobial agents belonging to nine different classes were tested: (1) penicillins: penicillin G (P, 1 U), ampicillin (AMP, 2 μg), and amoxicillin-clavulanic acid (AMC, 20/10 μg); (2) carbapenems: meropenem (MEM, 10 μg); (3) aminoglycosides: gentamicin (CN, 10 μg); (4) glycopeptides: vancomycin (VA, 30 μg); (5) tetracyclines: tetracycline (TE, 30 μg); (6) fluoroquinolones: ciprofloxacin (CIP, 5 μg); (7) phenicols: chloramphenicol (C, 30 μg); (8) macrolides: erythromycin (E, 15 μg); and (9) sulfonamides: sulphamethoxazole/trimethoprim (STX, 23.75/1.25 μg). The susceptibility of the isolates to antimicrobial agents was categorized (as susceptible or resistant) through a measurement of the inhibition zone. The tests were performed in triplicate, and the mean diameter of the inhibitory zones was calculated. For penicillin G, ampicillin, meropenem, erythromycin, sulfamethoxazole-trimethoprim, and amoxicillin-clavulanic acid, clinical breakpoints for *L. monocytogenes* susceptibility testing defined by the European Committee on Antimicrobial Susceptibility Testing (EUCAST) were used [31]. For vancomycin and tetracycline, the criteria for *Streptococcus pneumoniae* were adopted [31]. The criteria for staphylococci were adopted for ciprofloxacin, chloramphenicol, and gentamicin [32]. *Streptococcus pneumoniae* strain ATCC 49619 was used as the reference strain (quality control).

### 2.6. DNA Isolation

DNA isolation was performed with the use of a GeneMATRIX Bacterial & Yeast Genomic DNA Purification Kit (EURx, Gdańsk, Poland), according to the manufacturer’s recommendations.

### 2.7. Molecular Serotyping

The molecular serotyping of *L. monocytogenes* isolates was performed by multiplex PCR according to the method reported by Doumith et al. [33] and D’agostino et al. [34] with the use of six specific primers (*lmo0737*, *lmo1118*, *ORF2819*, *ORF2110*, *prs*, and *prfA*) (Table 1). The PCR was performed in a thermal cycler (BioRad, Madrid, Spain), and conditions for the genes amplification were as follows: 3 min of initial denaturation at 94 °C, followed by 35 amplification cycles of denaturation at 94 °C for 35 s, hybridization at 55 °C for 50 s, and an extension step at 72 °C for 60 s, ending with a final extension period of 72 °C for 7 min. The amplicons were separated by electrophoresis on 1.5% agarose gel in TAE buffer and visualized by ethidium bromide staining (EURx, Gdańsk, Poland). To estimate the size of the amplicons, a DNA Ladder in the range of 100–1000 bp was used (A&A Biotechnology, Gdansk, Poland). The proposed multiplex PCR profiling scheme does not distinguish within the *L. monocytogenes* species serovars 1/2a from 3a, 1/2c from 3c, 1/2b from 3b and 7, or 4b from 4d and 4e. This multiplex PCR scheme also does not distinguish serovars 4a and 4c of *L. monocytogenes* from other *Listeria* species.

### 2.8. Detection of Virulence Genes

All *L. monocytogenes* isolates were screened for virulence genes with several PCR assays, detecting nine specific (*inlA*, *inlC*, *inlJ*, *llsX*, *hlyA*, *lmo2672*, *plcA*, *plcB*, and *prfA*) genes [22,25,35,36,37] (Table 2).

The PCR conditions for the gene amplification were as follows: 4 min of initial denaturation at 94 °C, followed by 35 amplification cycles of denaturation at 94 °C for 30 s, hybridization at various temperatures depending on the primer sequence for 30 s, and an extension step at 72 °C for 2 min, ending with a final extension period of 72 °C for 10 min. The annealing temperature for genes *inlA*, *inlC*, *inlJ*, and *plcB* was 55 °C, for genes *llsX* and *hlyA*, it was 60 °C; for gene *lmo2672*, it was 58 °C; and for genes *plcA* and *prfA*, it was 50 °C (Table 2).

### 2.9. Pulsed-Field Gel Electrophoresis (PFGE) Typing

Pulsed-field gel electrophoresis (PFGE) was performed according to the protocol of the European Union Reference Laboratory for *L. monocytogenes* (ANSES, Maisons-Alfort, France) with the use of *Asc*I and *Apa*I restriction enzymes [38]. Genomic DNA was restricted with *Asc*I (5 UI per strain, 37 °C for 4 h) and *Apa*I (10 UI per strain, 37 °C for 4 h) (ThermoScientific, Abo, Poland). *L. monocytogenes* strain ATCC 19115 was used as the reference strain and digested with the same restriction enzymes. DNA fragments were separated on a CHEF DR-III system (BioRad Laboratories, Inc., Hercules, CA, USA). The gels were stained with 0.5 μg mL^−1^ of ethidium bromide for 15 min and photographed under UV transillumination using the QuantityOne (BioRad, Madrid, Spain) software v. 4.6 and GelDoc 2000 (BioRad, Madrid, Spain) system. The banding patterns were analyzed with bionumerics Gel Compar II 6.5 software (Applied Maths, Sint-Martens-Latem, Belgium) using the Dice coefficient with a position tolerance of 1.0%, an average optimization value of 1.0%, and the UPGMA (Unweighted Pair-Group Method with Arithmetic mean) algorithm.

## 3. Results

### 3.1. Patient Profile and Characteristics of Clinical Listeria monocytogenes Strains

Clinical strains were from patients with bloodstream infection over the age of 52 (mean 70) and neuroinfection in a child aged 10 years. Seven strains were isolated from venous blood cultures and one from cerebrospinal fluid cultures (Table 3). All patients with *Listeria* bloodstream infection had multiple comorbidities. In three cases (i.e., patients aged 53, 82, and 83), despite the treatment, patients died. In none of the patients was it possible to link the infection to the consumption of contaminated food. Detailed characteristics of each of the eight patients are presented in Table S1 (Supplementary Materials).

### 3.2. Listeria from Food Products

Out of 124 food samples, 7 of them were positive for rods belonging to the *Listeria* genus, of which 4 showed the presence of *L. monocytogenes* (3.2%) and 3 that of *Listeria innocua* (2.4%). *L. monocytogenes* strains were isolated from meat dumplings (*n* = 2), smoked Atlantic salmon (*n* = 1), and camembert cheese (*n* = 1) (Table 4). In this study, we also included 12 strains of *L. monocytogenes* collected from food products in 2020–2022 by NIPH-NIH and IAFB (Table 4).

A total of 24 strains of *L. monocytogenes* were included in further studies (8 clinical strains and 16 strains originating from food matrices).

### 3.3. Antimicrobial Susceptibility Testing, Molecular Serotyping, and Detection of Virulence Genes in Listeria monocytogenes Strains

All *L. monocytogenes* strains were susceptible to penicillin G, ampicillin, amoxicillin-clavulanic acid, meropenem, gentamicin, vancomycin, chloramphenicol, erythromycin, and sulfamethoxazole-trimethoprim. Eight strains isolated from food products were found to be resistant to ciprofloxacin (33.3%), and one food-based strain resistant to tetracycline (4.2%) was detected (Table 5). Clinical strains showed full sensitivity to all tested antibiotics.

Most of the tested isolates (*n* = 19) belonged to serotype 1/2a-3a (molecular group IIa) (79.2%); among clinical strains, it was 62.5% (*n* = 5), and among food-based strains, 87.5% (*n* = 14). The rest belonged to serotype 4ab-4b,4d-4e (molecular group IVb) (20.8%, *n* = 5), including 37.5% (*n* = 3) among clinical strains and 12.5% (*n* = 2) among food-based strains. None of the strains belonged to serotype 1/2c-3c (molecular group IIc), serotype 1/2b-3b-7 (molecular group IIb), or serotype 4a-4c (molecular group IVa) (Table 6).

The isolates were assessed for the detection of nine virulence-associated genes. Five virulence genes (*prfA*, *hlyA*, *plcB*, *inlA*, and *lmo2672*) were detected in all strains studied. The *plcA* gene was present in 87.5% of clinical strains and 93.75% of food-based strains, while the *inlC* and *inlJ* genes were detected in 100% of clinical strains and 93.75% or 56.25% of food-based strains, respectively. The *llsX* gene was the least common, in 37.5% of clinical strains and 18.75% of strains isolated from food products (Table 6).

**Figure 1:**
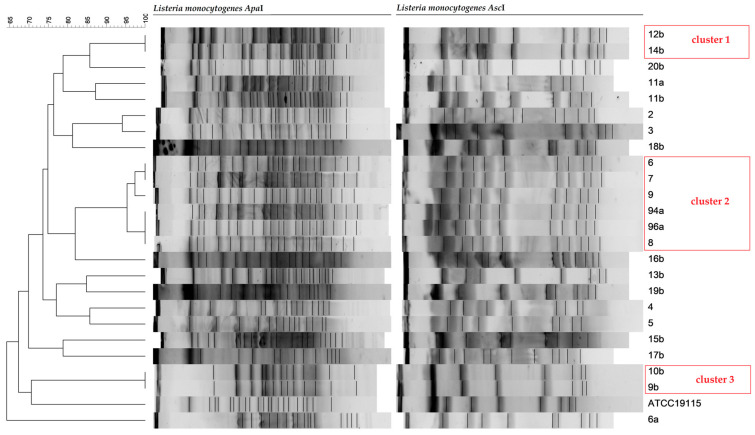
Dendrogram displaying PFGE profiles of *Listeria monocytogenes* isolates using two restriction enzymes: *Apa*I (**left side**) and *Asc*I (**right side**). The numeric and letter designations refer to the tested *L. monocytogenes* strains. *L. monocytogenes* strain ATCC 19,115 was used as the reference strain. Strains belonging to one cluster are marked with a red frame.

The genetic fingerprint of the twenty-four *L. monocytogenes* isolates is shown in Figure 1. Among the analyzed strains, 13 strains displayed unique PFGE profiles. The other 11 strains belong to 3 clusters of pulsotypes: cluster No. 1 (2 strains), cluster No. 2 (6 strains), and cluster No. 3 (2 strains). In the cases of *L. monocytogenes* strain 9b and 10b, they came from one production factory. Strains 12b and 14b came from food products from NIPH-NIH culture collection. Pulsotype 2 included four clinical strains isolated from patients in 2021 and 2022 and two strains cultured from food products in 2023.

## 4. Discussion

Listeriosis notification in humans is mandatory across all European Union/European Economic Area (EU/EEA) countries. Between 2018 and 2022, the number of listeriosis cases reported by consistently reporting EU/EEA countries declined from 2018 to 2020, followed by an increase in 2021 and 2022. In 2022, 30 countries reported 2770 confirmed cases of listeriosis within the EU/EEA [8]. In Poland, this increase continued in 2023, with 100 more cases recorded than in 2022 [9]. Although it is not the main pathogen causing foodborne infections, infections are often serious, requiring hospitalization and relatively often ending in death, especially in the elderly group. In Europe, *L. monocytogenes* invasive infections were most commonly reported in the age group over 64, in Poland; according to the Księżak and Sadkowska-Todys study [39], 65.5% of patients were over 60 years of age, and the most common form of invasive disease was BSI (49.1%) [39]. Our current research confirms that most cases of listeriosis involve older patients with comorbidities—the average age of patients with BSI was 70 years—and the three patients who died were 53, 82, and 83 years old. Among the comorbidities in patients with isolated *L. monocytogenes*, the most common is cancer (32.5%) [39]. In Polish research conducted by Kuch and co-workers [21], a high fatality rate was recorded, higher than in Europe, 40% (where it is 18.1%); in our study, it was 37.5%, but we had a small number of patients here [21]. In turn, in other Polish studies, Księżak and Sadkowska-Todys [39] present a mortality rate of 38.4% in 2012–2019, but in 2021, it was 20.83% [39]. The Polish population (like the European one) is aging: there are more people with chronic diseases, and an increasing number of people exposed to severe forms of *L. monocytogenes* infections can be expected, as well as associated hospitalization rates and uncertain therapy outcomes [40].

Invasive listeriosis always requires effective antibiotic therapy, in which ampicillin is used in the first line, often in combination with gentamicin [41], meropenem, or other antibiotics like rifampicin, vancomycin, or linezolid [41,42]. Antibiotic resistance in clinical strains of *L. monocytogenes* has been rarely reported so far, but when *Listeria* strains are exposed to sublethal concentrations of antimicrobials, they may develop resistance and pose a therapeutic problem [10]. Kuch and co-workers [21], who tested 344 strains of invasive infections, showed full sensitivity to 10 antibiotics of all tested strains [21]. Skowron and co-workers [13] detected resistance to erythromycin among strains isolated from blood and to penicillin among strains isolated from milk. 

The resistance of *Listeria* strains isolated from food is slightly higher. Lachtara and co-workers [43] tested the resistance of food and environmental strains to 17 antibiotics and described strains resistant primarily to ceftriaxone (37.8%), ceftriaxone and oxacillin (28.9%), and ceftriaxone and oxacillin and clindamycin (10.8%), but in the case of clinically important antibiotics such as ampicillin and gentamicin, all strains were susceptible. Single strains were resistant to ciprofloxacin, linezolid, tetracycline, or erythromycin [43]. High resistance rates (85%) of *L. monocytogenes* strains to clindamycin were also described in Poland by Wiśniewski and co-workers [44]. Maćkiw and co-workers [16] tested *Listeria* from RTE meat products and showed 83% resistance to ampicillin, which already seems very disturbing [16].

Our studies are comparable to other observations and confirm that clinical strains showed full sensitivity; resistance was detected among food strains, and it concerned ciprofloxacin (33.3% of strains) and tetracycline (4.2% of strains).

In the years 2016–2021, numerous works were published in Poland precisely characterizing the molecular strains of *L. monocytogenes* from food products from various food groups: meat, fish, vegetables, and RTE products. *L. monocytogenes* was most often diagnosed in fish and fish products, meat, dairy products, and RTE products [43,45,46]. Szymczak and co-workers [46] indicated the presence of *L. monocytogenes* in as many as 13.5% of the tested Polish RTE products, most of the strains belonged to serotype 4ab-4b-4d-4e. *L. monocytogenes* was most often isolated from dumplings with meat (53%) and croquettes and meat (45%). Two of our strains isolated in this study came from dumplings with meat, one from salmon and one from Camembert cheese [46]. Skowron and co-workers [13] estimated the level of *L. monocytogenes* in meat at 2.1% [13]. Another study, conducted by Maćkiw and co-workers [16], which tested RTE and RTE meat products as part of the national official control program at retail indicated *L. monocytogenes* contamination at the level of 0.1% for RTE food products and 0.3% for RTE meat products; most of the strains belonged to serotype 4ab-4b-4d-4e, and in RTE meat products, up to 1/2a-3a [16]. In turn, in research conducted by Maćkiw and co-workers [15] on vegetable samples available in retail, the presence of *L. monocytogenes* was at a level of 0.56%, and most of the strains were from serotype 1/2a-3a [15]. In 2017, Wieczorek and Osek [47] analyzed fresh and smoked fish and indicated the presence of *L. monocytogenes* in 18.9% of products, and molecular tests showed a large dominance of isolates from group IIa (1/2a-3a) (96.4%) [47].

Most clinical *L. monocytogenes* strains were tested by Kuch and co-workers [21], and they came from invasive listeriosis from 1997 to 2013. Research has shown that most clinical isolates came from BSI and belong to serogroup 4b. The strains did not originate from any proven epidemic. Moreover, among clinical strains, they have recorded a high, higher than in our studies, predominance of serogroup 1/2a [21].

PFGE subtyping obtained with combined analysis using *Asc*I and *Apa*I enzymes for 24 *L. monocytogenes* isolates yielded 13 different PFGE restriction profiles. The remaining 11 strains belonged to 3 clusters: pulsotype 1 (strains: 12b and 14b), pulsotype 2 (strains: 6, 7, 8, 9, 94a, and 96a), and pulsotype 3 (strains: 9b and 10b). All strains included in the three pulsotypes belonged to molecular group IIa. Pulsotypes 1 and 3 collected isolates only from food samples. In the cases of *L. monocytogenes* strain 9b (KKP 3270) and *L. monocytogenes* strain 10b (KKP 3271) (from IAFB culture collection), they came from one production factory, so there is a high probability that the strain isolated from sushi (*L. monocytogenes* strain 9b- KKP 3270) came from raw salmon (*L. monocytogenes* strain 10b -KKP 3271). These strains could also come from food production areas, as *L. monocytogenes* is a serious problem in food production factories, mainly due to its ability to form persistent biofilms. The genetic similarity of these strains was confirmed by PFGE (pulsotype 3). In the case of *L. monocytogenes* strain 12b (7045C) (from NIPH-NIH culture collection), it was isolated from minimally processed food product, so there is a high probability that its source was raw materials (environment) (pulsotype 1). However, pulsotype 2 shared six isolates both isolated from humans (strains 6, 7, 8, and 9) and food products (96a from camembert cheese and 94a from smoked salmon), which were additionally from different years. Among pulsotype 2 strains, three strains isolated from patients were in 2021, one strain isolated from patient was in 2022, and two strains isolated from food products were in 2023. The similarity of human and food strain profiles can reveal epidemiological relatedness. All strains were from the field of Krakow, which may indicate the presence of specific types of strains in the environment in the region. However, this will require further research.

Knowledge not only about resistance or serogroup but also about the pathogenic and adaptive nature of *L. monocytogenes*, especially relating to virulence genes and phenotypic features of strains, may be useful for assessing the risk of infections by strains isolated from food [48]. The virulence of *L. monocytogenes* strains is associated with numerous genes and proteins. Certain genes are grouped within genomic and pathogenicity islands, with LIPI-1 and LIPI-3 harboring those most closely associated with the infectious life cycle [49]. In our study, we detected mainly genes from the LIPI-1 group involved in the intracellular infection cycle of *L. monocytogenes*; 100% of the strains carried the *hlyA*, *prfA*, *plcB*, *inlA*, and *lmo2672* genes. Many European and Polish studies indicate that virulence genes from pathogenicity islands LIPI-1 are present in most strains isolated from food products [44,47,50,51,52,53,54].

The least common gene was *llsX*—one of the genes from the listeriolysin L (LLS) operon—and we diagnosed it slightly more often among clinical strains (37.5% vs. 18.75% in food products). The greatest difference between clinical and food strains concerned the presence of the internalin J (*inlJ*) gene; it was present in 100% of clinical strains and 56.25% of food-based strains. Maćkiw and co-workers [15] also showed a lower incidence of the *inlJ* gene (82%) and the *llsX* gene (22%) in the *L. monocytogenes* strains isolated from vegetables [15].

There are significant differences in gene expression between planktonic *L. monocytogenes* and biofilm-forming cells regarding the expression of internalin A and C (*inlA* and *inlC*), *prfA* activator, and listeriosin O (*hlyA*) [54,55,56]. Mutations in the *inlA* gene, which are more common among food strains, cause *L. monocytogenes* to have an increased ability to form biofilms but a lower virulence potential [57]. In contrast, *prfA*-negative mutants are unable to form biofilms at later stages [58].

Highly pathogenic strains may be associated with *L. monocytogenes* outbreaks. Most of the large epidemics described in Europe were related to the consumption of fish, including cold smoked salmon and meat products [59]. In turn, the CDC has recorded interstate epidemics in recent years related to cheese, dairy products including ice cream, and fruits and vegetables [60].

In 2022, 22 such epidemics were recorded in European countries [7]. No epidemics have been reported in Poland in recent years, perhaps due to difficulties in determining the source of infection and establishing connections between cases, as well as in epidemiological investigations using typing methods that are not always carried out [21].

ECDC and EFSA are working together to quickly identify outbreaks. In 2022, the EFSA One Health WGS system became available to Member States to exchange cgMLST (core genome multi-locus sequence typing) profiles and minimum metadata. WGS allows you to effectively type epidemic strains and quickly and accurately identify clusters in both local and international epidemics [17]. Unfortunately, in Poland it is not yet a method used routinely during epidemiological investigations; it is more often used during scientific research. In the case of clinical isolates, Kuch and co-workers [21] showed dominant CC6 and CC1 clones [21]. Food-based *L. monocytogenes* strains in research conducted by Lachtara and co-workers [43] mainly belonged to CC155, CC121, and CC8. In our study, we did not use the WGS technique; we only used the PFGE method using two restriction enzymes and confirmed the presence of three clusters. 

Therefore, it seems that the diagnosis of infection in humans should be associated with a more detailed interview of the patient to determine the source of infection, as well as with a thorough molecular examination of strains to assess their serotype, virulence genes, and typing, which would facilitate the identification of *L. monocytogenes* clones occurring in Poland.

Integrating epidemiological data from humans, animals, and food with molecular and genotypic information offers a powerful approach to gaining a deeper understanding of this pathogen’s ecology throughout various stages of the food chain. There is also a need to raise awareness among doctors, consumers, and food producers about the potential presence of *L. monocytogenes* in food and the growing number of infections, especially among the elderly and people at risk. At the international level, the Joint FAO/WHO Expert Meetings on Microbial Risk Assessment (JEMRA) has recommended expanding risk assessment to *L. monocytogenes* in RTE food products, taking a perspective from primary production to consumption and reviewing susceptible population groups [61].

### Limitation

In this study, relatively few clinical strains were examined, and they came from a single center. We did not use sequencing methods for genotyping.

## 5. Conclusions

This study highlights the serious nature of listeriosis, particularly within the aging population in Poland, where the rate of hospitalizations and mortality remains high. The findings underscore the predominance of serotypes 1/2a in food samples and both 1/2a and 4b in clinical samples, reflecting consistent trends in the *Listeria monocytogenes* distribution. Despite comprehensive surveillance, clinical strains in Poland are less frequently subjected to detailed molecular testing compared to food-derived strains, complicating the identification of infection sources. This research also underscores the concerning emergence of antibiotic resistance among *L. monocytogenes* strains isolated from food, particularly resistance to ciprofloxacin and tetracycline, which could pose significant therapeutic challenges. This study advocates for the enhanced molecular surveillance of clinical strains and the integration of epidemiological data to better understand the transmission dynamics of *L. monocytogenes* and mitigate its public health impact.

## Figures and Tables

**Table 1:** The primer pairs used for molecular serotyping of *L. monocytogenes* strains.

Target Gene	Primer Sequences 5′–3′	Product Size	Serovar Specificity	Protein Encoded by the Target Gene	Reference
* lmo0737 *	F–AGGGCTTCAAGGACTTACCC R–ACGATTTCTGCTTGCCATTC	691 bp	*L. monocytogenes* serovars 1/2a, 1/2c, 3a, and 3c	hypothetical protein belonging to a member of TIM phosphate binding superfamily	[33]
* lmo1118 *	F–AGGGGTCTTAAATCCTGGAA R–CGGCTTGTTCGGCATACTTA	906 bp	*L. monocytogenes* serovars 1/2c and 3c	domain-containing protein	[33]
* ORF2819 *	F–AGCAAAATGCCAAAACTCGT R–CATCACTAAAGCCTCCCATTG	471 bp	*L. monocytogenes* serovars 1/2b, 3b, 4b, 4d, and 4e	putative transcriptional regulator	[33]
* ORF2110 *	F–AGTGGACAATTGATTGGTGAA R–CATCCATCCCTTACTTTGGAC	597 bp	*L. monocytogenes* serovars 4b, 4d, and 4e	putative secreted protein	[33]
* prs *	F–GCTGAAGAGATTGCGAAAGAAG R–CAAAGAAACCTTGGATTTGCGG	370 bp	All *Listeria* species	putative phosphoribosyl pyrophosphate synthetase	[33]
* prfA *	F–GATACAGAAACATCGGTTGGC R–GTGTAACTTGATGCCATCAGG	274 bp	All *L. monocytogenes* strains	central virulence gene regulator	[34]

**Table 2:** Primers used for the detection of virulence genes in *L. monocytogenes* strains.

Target Gene	Primer Sequences 5′–3′	Annealing Temperature	Product Size	Protein Encoded by the Target Gene	Reference
* inlA *	F–ACGAGTAACGGGACAAATGC R–CCCGACAGTGGTGCTAGATT	55 °C	800 bp	internalin A	[22]
* inlC *	F–AATTCCCACAGGACACAACC R–CGGGAATGCAATTTTTCACTA	55 °C	517 bp	internalin C	[22]
* inlJ *	F–TGTAACCCCGCTTACACAGTT R–AGCGGCTTGGCAGTCTAATA	55 °C	238 bp	internalin J	[22]
* llsX *	F–TTATTGCATCAATTGTTCTAGGG R–TTATTGCATCAATTGTTCTAGGG	60 °C	200 bp	listeriolysin S	[25]
* hlyA *	F–GTTAATGAACCTACAAGACCTTCC R–ACCGTTCTCCACCATTCCCA	60 °C	707 bp	listeriolysin O	[25]
* lmo2672 *	F–CGGCACACTTGGATTCTCAT R–AGGGCTAGTGACGGATGCTA	58 °C	481 bp	transcriptional regulator	[35]
* plcA *	F–TCCCATTAGGTGGAAAAGCA R–CGGGGAAGTCCATGATTAGA	50 °C	840 bp	phosphatidyl inositol phospholipase C	[36]
* plcB *	F–CAGCTCCGCATGATATTGAC R–CTGCCAAAGTTTGCTGTGAA	55 °C	723 bp	phosphatidyl choline phospho-lipase C	[36]
*prfA*	F–AACGGGATAAAACCAAAACCA R–TGCGATGCCACTTGAATATC	50 °C	469 bp	transcriptional factor	[37]

**Table 3:** Source of origin and year of isolation of all collected clinical *Listeria monocytogenes* strains.

Strain Number	Original Number	Clinical Material	Species Identification According to VITEK MS	Patient Age, Sex	Year of Isolation
2	PB9156	Cerebrospinal fluid	* Listeria monocytogenes *	10, M	2019
3	185bak21	Venous blood	* Listeria monocytogenes *	58, M	2021
4	50322bak22	Venous blood	* Listeria monocytogenes *	53, F	2022
5	26560bak22	Venous blood	* Listeria monocytogenes *	52, F	2022
6	67648bak21	Venous blood	* Listeria monocytogenes *	91, F	2021
7	53453bak21	Venous blood	* Listeria monocytogenes *	82, F	2021
8	66589bak21	Venous blood	* Listeria monocytogenes *	83, F	2021
9	30340bak22	Venous blood	* Listeria monocytogenes *	73, M	2022

**Table 4:** Source of origin and year of isolation of all collected food-based *Listeria* strains.

Strain Number	Original Number	Food Products	Species Identification According to VITEK MS	Year of Isolation
Strains isolated from food products in this study (*n* = 7)				
6a	This study	Meat dumplings	* Listeria monocytogenes *	2023
11a	This study	Meat dumplings	* Listeria monocytogenes *	2023
31a	This study	Smoked salmon	* Listeria innocua *	2023
38a	This study	Smoked salmon	* Listeria innocua *	2023
73a	This study	Pielmieni	* Listeria innocua *	2023
94a	This study	Smoked Atlantic salmon	* Listeria monocytogenes *	2023
96a	This study	Camembert cheese	* Listeria monocytogenes *	2023
Strains from NIPH-NIH collection (*n* = 10)				
11b	6982C	Smoked Atlantic salmon	* Listeria monocytogenes *	2022
12b	7045C	Tatar sausage (raw sausage made from ground meat)	* Listeria monocytogenes *	2022
13b	7117	Frozen potato dumplings	* Listeria monocytogenes *	2022
14b	7197	Meat dumplings	* Listeria monocytogenes *	2022
15b	6780	Smoked salmon trout	* Listeria monocytogenes *	2022
16b	6797E	Ham sausages	* Listeria monocytogenes *	2022
17b	6556C	Mixed salad	* Listeria monocytogenes *	2022
18b	6535	Onion-flavored tatar sausage	* Listeria monocytogenes *	2022
19b	6310	Potato and cheese dumplings	* Listeria monocytogenes *	2022
20b	6317	Cabbage and carrots mix	* Listeria monocytogenes *	2022
Strains from IAFB collection (*n* = 2)				
9b	KKP 3270	Sushi	* Listeria monocytogenes *	2020
10b	KKP 3271	Raw salmon	* Listeria monocytogenes *	2020

Abbreviation: NIPH-NIH—National Institute of Public Health—National Institute of Hygiene—National Research Institute; IAFB—Prof. Wacław Dąbrowski Institute of Agricultural and Food Biotechnology—State Research Institute.

**Table 5:** Phenotype resistance of *L. monocytogenes* strains.

*Listeria monocytogenes* Strain Number	Phenotypic Antibiotic Resistance Pattern
9b	CIP
10b	CIP
11b	TE-CIP
12b	CIP
14b	CIP
16b	CIP
17b	CIP
20b	CIP

Notes: TE—tetracycline; CIP—ciprofloxacin.

**Table 6:** Serotypes and presence of virulence genes in clinical and food-based *Listeria monocytogenes* strains.

	Strain Number	Serotype (Molecular Group)	Virulence-Associated Genes								
			* prfA *	* hlyA *	* plcB *	* plcA *	* inlA *	* inlC *	* inlJ *	* lmo2672 *	* llsX *
Clinical strains	2	4ab-4b,4d-4e/(IVb)									
	3	4ab-4b,4d-4e/(IVb)									
	4	1/2a-3a (IIa)									
	5	4ab-4b,4d-4e/(IVb)									
	6	1/2a-3a (IIa)									
	7	1/2a-3a (IIa)									
	8	1/2a-3a (IIa)									
	9	1/2a-3a (IIa)									
Total (%)			100	100	100	87.5	100	100	100	100	37.5
Food strains	6a	1/2a-3a (IIa)									
	11a	4ab-4b,4d-4e/(IVb)									
	94a	1/2a-3a (IIa)									
	96a	1/2a-3a (IIa)									
	9b	1/2a-3a (IIa)									
	10b	1/2a-3a (IIa)									
	11b	4ab-4b,4d-4e/(IVb)									
	12b	1/2a-3a (IIa)									
	13b	1/2a-3a (IIa)									
	14b	1/2a-3a (IIa)									
	15b	1/2a-3a (IIa)									
	16b	1/2a-3a (IIa)									
	17b	1/2a-3a (IIa)									
	18b	1/2a-3a (IIa)									
	19b	1/2a-3a (IIa)									
	20b	1/2a-3a (IIa)									
Total (%)			100	100	100	93.75	100	93.75	56.25	100	18.75

Abbreviation: gray field—gene present; white field—gene absent.

## Data Availability

The original contributions presented in the study are included in the article/Supplementary Material, further inquiries can be directed to the corresponding author/s.

## Supplementary Materials

The following supporting information can be downloaded at https://www.mdpi.com/article/10.3390/pathogens13090725/s1: Table S1: Characteristics of patients from whom the *L. monocytogenes* strains used in the study were derived.
