# Supplementary material for: Genetic Diversity, Virulence Factors and Antibiotic Resistance of Listeria monocytogenes from Food and Clinical Samples in Southern Poland
Source: Pathogens. 2024 Aug 27;13(9):725. doi: 10.3390/pathogens13090725 (PMC11434881; doi:10.3390/pathogens13090725)
Supplement: Supplementary file 1 [file pathogens-13-00725-s001.zip › pathogens-3168374-supplementary.pdf]

Table S1. Characteristics of patients from whom the *L. monocytogenes* strains used in the study were derived.

**Strain No. 2**	The strain was cultured from the cerebrospinal fluid of a 10-year-old male patient admitted with suspicion of neuroinfection (after a head injury).
**Strain No. 3**	The strain was isolated from a 53-year-old man admitted to the ICU due to acute renal failure in the course of acute pancreatitis. The patient was in a serious condition, confused, and in respiratory failure. In the interview, the patient had numerous medical problems: alcohol addiction syndrome, reported abdominal pain, nausea, and vomiting. Broad-spectrum empiric therapy was changed to ampicillin-gentamicin. Despite intensive treatment, there was no improvement in the clinical condition, and the patient died due to multi-organ failure 3 weeks after admission.
**Strain No. 4**	The strain was isolated from a 53-year-old woman admitted to the Department of Surgery from the Emergency Department. The patient complained of shortness of breath, chest pain, palpitations, and weakness after the last cycle of chemotherapy. Pulmonary embolism was diagnosed. She had a history of breast cancer with metastases in the bones and liver. The treatment included ampicillin in combination with meropenem and linezolid. The patient was discharged home in optimal condition, which is adequate for the comorbidities.
**Strain No. 5**	The strain was isolated from a 52-year-old patient from the nephrology department suffering from systemic lupus erythematosus in the form of nephropathy. On admission, she reported symptoms of uremia and weakness. *L. monocytogenes* was cultured from blood culture. The patient was discharged home in good condition, with a recommendation for peritoneal dialysis in the future.
**Strain No. 6**	The strain came from a 91-year-old patient undergoing chronic treatment for hypertension, hypercholesterolemia, and osteoarthritis. She was admitted to the geriatrics ward when she was found lying in her apartment; her general condition was average. The treatment included intravenous hydration and empirical antibiotic therapy with ceftriaxone, and in the following days, the growth of *L. monocytogenes* was detected in a blood culture. Antibiotic therapy was modified to ampicillin and gentamicin for 21 days. After treatment, the patient was transferred to the COVID ward due to a positive swab for SARS-CoV-2 virus infection.
**Strain No. 7**	The strain came from a blood culture from an 82-year-old patient admitted to the intensive care unit from the surgical department after surgery due to intestinal obstruction with symptoms of anuria and increasing parameters of inflammation. The history of hypertension, type 2 diabetes, and paroxysmal atrial fibrillation were noted. Multidisciplinary, integrated treatment was implemented, including ceftriaxone and metronidazole. The measures were ineffective, and the patient died.
**Strain No. 8**	The strain was isolated from an 83-year-old patient with hypertension, hypothyroidism, and cholelithiasis admitted due to high fever, dehydration, and neurological disorders. Their empirical treatment included broad-spectrum antibiotics. *L. monocytogenes* was recovered from blood culture. Despite treatment, the patient's condition deteriorated, and respiratory failure deepened. The patient died as a result of multi-organ failure.
**Strain No. 9**	The strain was isolated from a 73-year-old man admitted to the Department of Metabolic Diseases and Diabetology after reporting to the Emergency Department due to fever, weakness, polyuria, and high-inflammatory parameters. He had a history of myeloproliferative neoplasm, and the patient was also a repeated recipient of blood products. *L. monocytogenes* and *Staphylococcus aureus* were cultured in blood cultures, and *Enterococcus faecalis* HLAR (high-level aminoglycoside resistance) was cultured in urine culture. Treatment with ampicillin, vancomycin, and metronidazole was initiated due to focal changes in the liver visible on ultrasound. Improvement was achieved, and the patient was discharged home.
